# Stability of Proximal Femoral Osteotomies in Pediatric Bone Models Fixed with Flexible Intramedullary Nails and Evaluated by the Finite Element Method

**DOI:** 10.1055/s-0044-1785467

**Published:** 2024-04-10

**Authors:** Mário Augusto Ferreira Cruz, José Vinícius Lima Santana, Leonardo Rigobello Battaglion, José Batista Volpon

**Affiliations:** 1 Universidade Tiradentes, Aracaju, Sergipe, Brasil; 2 Laboratório de Bioengenharia, Faculdade de Medicina de Ribeirão Preto, Universidade de São Paulo (FMRP-USP), Ribeirão Preto, São Paulo, Brasil; 3 Departamento de Ortopedia e Anestesiologia, Faculdade de Medicina de Ribeirão Preto, Universidade de São Paulo (FMRP-USP), Ribeirão Preto, São Paulo, Brasil

**Keywords:** femoral fractures, finite element analysis, fracture fixation, intramedullary

## Abstract

**Objective** To evaluate the stability of osteotomies created in the subtrochanteric and trochanteric regions in a pediatric femur model fixed by flexible intramedullary rods.

**Method** Tomographic sections were obtained from a pediatric femur model with two elastic titanium rods and converted to a three-dimensional model. This model created a mesh with tetrahedral elements according to the finite element method. Three virtual models were obtained, and osteotomies were performed in different regions: mediodiaphyseal, subtrochanteric, and trochanteric. A vertical load of 85N was applied to the top of the femoral head, obtaining the displacements, the maximum and minimum main stress, and the equivalent Von Mises stress on the implant.

**Results** With the applied load, displacements were observed at the osteotomy site of 0.04 mm in the diaphyseal group, 0.5 mm in the subtrochanteric group, and 0.06 mm in the trochanteric group. The maximum stress in the diaphyseal, subtrochanteric, and trochanteric groups was 10.4 Pa, 7.52 Pa, and 26.4 Pa, respectively. That is around 40% higher in the trochanteric group in regards to the diaphyseal (control). The minimum stress of the bone was located in the inner cortical of the femur. The equivalent Von Mises stress on the implants occurred at osteotomy, with a maximum value of 27.6 Pa in the trochanteric group.

**Conclusion** In both trochanteric and subtrochanteric osteotomies, fixation stability was often lower than in the diaphyseal model, suggesting that flexible intramedullary nails are not suitable implants for proximal femoral fixations.

## Introduction

Intramedullary elastic fixation is a reliable and effective option for treating fractures in the pediatric femur's diaphysis. 1 2 However, when a fracture occurs in the trochanteric or subtrochanteric regions, the use of flexible rods is questioned due to possible insufficiency of mechanical stability to maintain the reduction and provide consolidation. 3 4 5 6 Under these conditions, it is interesting to simulate the mechanical behavior of an implant in order to anticipate whether it depends on clinical conditions.

There are usually two methods of evaluating the mechanical behavior of bone and implants: direct experimental techniques (or mechanical methods) and mathematical models. However, direct experimental techniques have disadvantages, being prone to errors and inaccuracies. 7

The Finite Element Method (FEM) is a powerful tool initially developed in the 1950s and widely accepted after investments in technology by the National Aeronautics and Space Administration (NASA). 8 In the field of Engineering, this method is used to solve conditions such as stress analysis, fluid flow, electromagnetism, and heat transfer using computer models. 8

In the Medical field, especially in Orthopedics and Biomechanics, the first records of the application of the FEM date back to the 1970s, when estimates of the ability of different types of tests to predict the mechanical behavior of bones were carried out. 7 9 Through the FEM, it is possible to accurately represent complex geometries and incorporate the different properties of materials, allowing the application of loads at specific points in the structure. This way, obtaining information about the maximum and minimum stress and deformations is possible. 7 Therefore, FEM is used to accurately predict the response of an implant when subjected to a variety of loads, in addition to incorporating the effect of the interfaces between the implant and the bone. 7 10 11

The objective of this study was to evaluate the stability provided by two flexible intramedullary rods in simulations of fractures located in the subtrochanteric, trochanteric, and diaphyseal regions created in a pediatric femur model using the finite element method.

## Material and Methods

This is a laboratory study using artificial bone models, and therefore, the Institutional Research Committee's approval of the project is waived.

An infant femur model with dimensions corresponding to a 9-year-old child (Sawbone Inc., Pacific Research Laboratories Inc., WA, United States). This synthetic bone has mechanical properties similar to human bone. 12 13

The preparation of the specimen was described earlier. 6 In summary, two flexible titanium rods ( *Titanium Elastic Nail* - TEN®, TiGa 114v, DePuy Synthes®, Oberdorf, Switzerland) with a diameter of 3.5 mm were inserted retrograde into the spinal canal. Radiography were performed to confirm proper positioning, followed by computed tomography of the entire bone model, archived in the DICOM communication protocol ( *Digital Imaging and Communications in Medicine* ). Computed tomography was completed using a Siemens ® 16-channel Tomograph, Emotion model (Erlangen, Germany), with a resolution of 512 × 512 and a cutting distance of 1.0 mm. DICOM was imported into the InVesalius® program (free software of the Renato Archer Information Technology Center, Campinas, São Paulo, Brazil), which enabled the generation of segmented models of the imported anatomical system for the three-dimensional (3D) construction of the anatomical structure. Once the volumetric object reconstructed in three dimensions was obtained, the software allowed the export of the file in the Standard Triangle Language (STL) format.

The Rhinoceros® 6 program (Robert McNeel & Associates, Seattle, WA, United States), version 6, generated virtual 3D models of each bone-stem set. To obtain a more accurate and faithful contour, we carried out reshuffles on the resulting intersection lines. These lines were drawn considering the region under study and may contain variations in the number of points according to the need for details of the area in question. Then, these lines were intersected and cut off, forming a set of three or four lines. This set allowed the generation of a three-dimensional surface.

The analysis by the FEM was conducted by the SimLab® program (HyperWorks, Troy, MI, USA), using the Optistruct solver.

To simulate the fractures, osteotomies were performed in the virtual models at three levels: cut at the level of the lesser trochanter (trochanteric group), cut located 3.5 cm distally to the lesser trochanter (subtrochanteric group), and cut in the central region of the diaphysis (mediodiaphyseal group, or control). Tetrahedral elements were used for knitting, and the number of knots was defined. In the virtual environment, a load of 85.0 N was applied to the top of the femoral head in the vertical direction, and the corresponding deformations and stresses were obtained.

For the simulations, it was necessary to know and define the material properties of each of the digital models' parts, namely cortical bone, spongy bone, and titanium alloy (TiGa114v). The properties of the materials used for the simulations are presented in Chart 1 .

**Chart 1:** Properties of materials used in simulations

Material	Properties	
	Modulus of Elasticity (MPa)	Poisson's Ratio (v)
Cortical bone	137	0.3
Cancellous bone	13.7	0.3
Titanium rod	114	0.33

## Results

With a loading of 85.0 N, the following displacements were obtained at the osteotomy simulation site: 0.04 mm in the control group, 0.5 mm in the subtrochanteric group, and 0.06 mm in the trochanteric group.

**Fig. 1:**
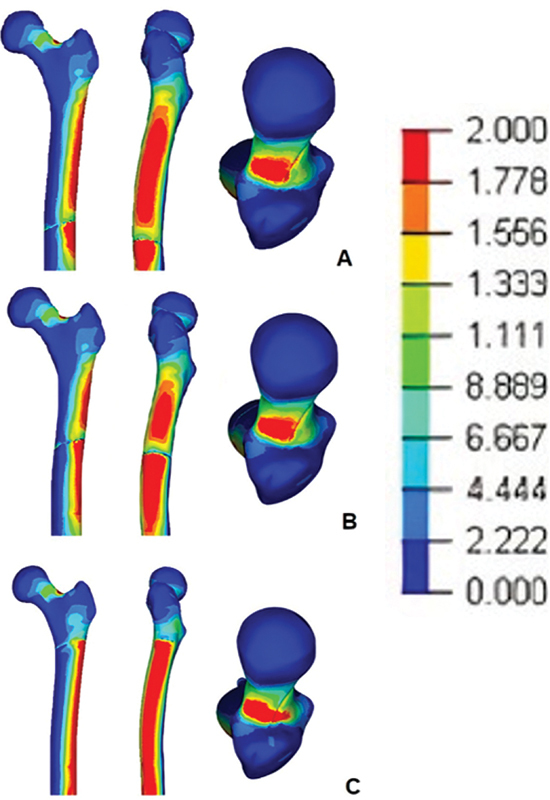
Distribution of the areas of maximum stress in the proximal regions of the femur in the simulations of the three types of osteotomies. A - Diaphyseal Osteotomy, B - Subtrochanteric Osteotomy, C - Trochanteric Osteotomy. The red colors represent the areas of greatest stress.

The greatest areas of stress were identified in the lateral cortical of the femur and the upper region of the neck. The main maximum stress reached 10.4 Pa, 7.52 Pa, and 26.4 Pa in the control, subtrochanteric, and trochanteric groups, respectively ( Fig. 1 ).

**Fig. 2:**
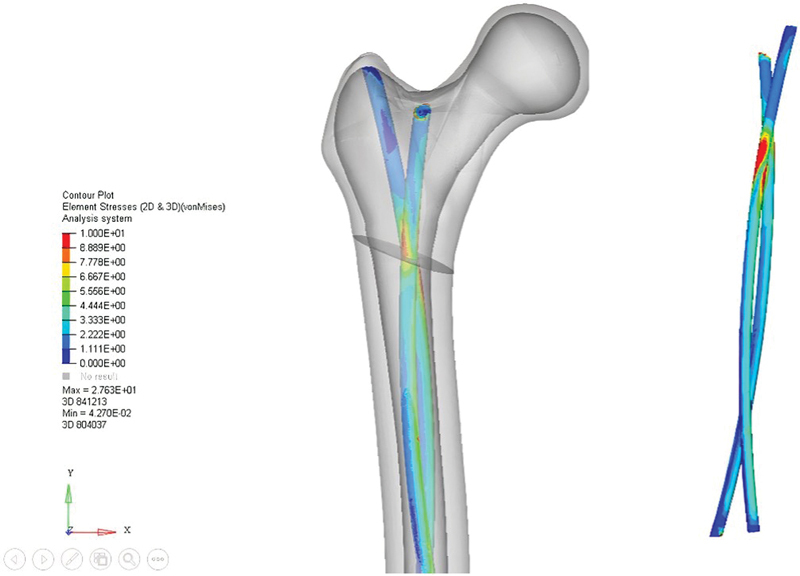
The figure represents the reconstruction of the proximal region of the femur, the osteotomy section, and the flexible rods. The rods without the bone contour are presented in detail on the side, illustrating the concentration of Von Mises equivalent stress higher in the osteotomy region (areas in red; critical region). If there is implant failure, it will occur at this level, leading to loss of reduction.

The main minimum stress face in the bone was identified in the medial cortical of the femur, presenting values of −11.6 Pa in the control group, -9.95 Pa in the subtrochanteric group, and −25.9 Pa in the trochanteric osteotomy. The equivalent Von Mises stress on the implants was observed in the osteotomy region, reaching a maximum value of 27.6 Pa in the trochanteric group ( Fig. 2 ).

## Discussion

Fractures in the subtrochanteric region of the femur have a strong tendency to deflect the proximal fragment in bending, varus, and external rotation, which is associated with shortening. 14 15 16 This results in increased stress between the fragments, which become more dependent on the stabilizing effect of the implant.

Therefore, the fixation must counteract the mechanical moments generated by local forces, providing adequate stability to maintain the reduction and allow consolidation. Thus, elastic rods may not meet these criteria, as already shown by clinical reports 3 and mechanical tests, 6 not being indicated for fractures in the most proximal regions.

To study the stability of the bone-implant model set, we used the FEM, used to simulate and verify the distribution of stress and displacements from the solution of equilibrium equations under loads. 17 To use the methodology, it was necessary to use a model of a fracture represented by an oblique osteotomy. The FEM provides the theoretical and mathematical substrates. However, in the case of fractures, it is applied to an idealized model. Therefore, it has the inconvenience of not taking into account many characteristics of the fracture, such as irregularities and different inclinations of the stroke, in addition to the possibility of presenting more than one fragment. In addition, it does not consider the action of soft parts in stabilizing/destabilizing the fracture. This limitation is inherent to the method; however, even with all the simplification, it is very useful in preclinical evaluations of implant development, for example, which is useful from the point of view of cost, time, and ethical research with human beings. Simplifications and restrictions also occur in studies in Engineering and other Exact Sciences.

There are several studies involving FEM in Orthopedics in the literature, and the topics involving fracture fixation and treatment of bone tumor lesions are the most addressed. 11 18 19 20 21 22 The FEM, because it is non-invasive, provides important biomechanical information, as well as assists in the development of orthopedic devices and has been more widely used in models of anatomical structures of adults, including for simulations of fixation of unstable subtrochanteric fractures. 20 23 24 Wang et al. 20 evaluated the biomechanical performance of three implants to treat unstable subtrochanteric fractures in adults using the FEM and observed that the proximal femoral stem was more stable than the blocked stem and the LISS system ( *Less Invasive Reverse System* ).

Our results showed that the most proximal osteotomy (trochanteric) presented the highest maximum stress and the highest Von Mises equivalent stress, which indicates that the implant's mechanical demand is higher in this site than in the other two groups. In addition, since the greatest Von Mises equivalent stress occurs at the sites of osteotomies, it is noticed that the implants serve as “tutors” and protect the fracture. This was also observed in the study by Soni et al. 25 , who performed a two-dimensional simulation of femoral fractures in children with the FEM to evaluate the effectiveness of using flexible rods constructed of steel or titanium.

In this study, when loading was applied, the regions of greatest stress were in the lateral cortical of the femur and the upper region of the neck. These results show that, with the load, the trochanteric cut presented a 153% higher stress request than the control (mediodiaphyseal cut).

However, the fragments' displacement at the osteotomy site was very small in all groups, which can be attributed to the low loading (85.0 N) applied to the systems. This value was selected after considering the mass of the unloaded lower limb of a 10-year-old child (∼8.5 kg) 26 ; therefore, intentional loading is not recommended clinically in the early postoperative phase. Additionally, this loading restricted the deformation to the elastic phase of the implants; that is, no irreversible deformation occurred in the clinic. If this limit is exceeded, there will be permanent deformation of the implant and loss of fracture reduction.

## Conclusions

For osteotomies in the trochanteric and subtrochanteric regions, there is greater mechanical demand for the implant, which may exceed the stabilization limits of the flexible intramedullary nails. Thus, clinically, this type of implant should be indicated in the classic situations for which it was designed, that is, in fractures of the diaphyseal region of the femur.
