# Supplementary material for: Stability of Proximal Femoral Osteotomies in Pediatric Bone Models Fixed with Flexible Intramedullary Nails and Evaluated by the Finite Element Method
Source: Rev Bras Ortop (Sao Paulo). 2024 Apr 10;59(2):e278–83. doi: 10.1055/s-0044-1785467 (PMC11006526; doi:10.1055/s-0044-1785467)
Supplement: Related texts

# Research article: Estabilidade de osteotomias proximais em modelos do fêmur pediátrico fixadas por hastes intramedulares flexíveis e avaliadas pelo método dos elementos finitos

**Keywords:** fraturas do fêmur, análise de elementos finitos, fixação intramedular de fraturas

## Abstract

**Objetivo** Avaliar a estabilidade de osteotomias criadas nas regiões subtrocantérica e trocantérica em modelo de fêmur pediátrico, fixadas por hastes intramedulares flexíveis.

**Método** A partir de um modelo de fêmur pediátrico com duas hastes elásticas de titânio, foram obtidos cortes tomográficos que foram convertidos para um modelo tridimensional. Neste modelo foi criado uma malha com elementos tetraédricos, de acordo com o método dos elementos finitos. Foram obtidos três modelos virtuais, e realizadas osteotomias em regiões diferentes: mediodiafisária, subtrocantérica e trocantérica. Foi aplicado um carregamento vertical de 85N no topo da cabeça do fêmur, obtidos os deslocamentos, a tensão máxima e mínima principal e tensão equivalente de Von Mises no implante.

**Resultados** Com o carregamento aplicado foram observados deslocamentos no local da osteotomia de 0,04mm no grupo diafisário, 0,5mm no subtrocantérico e 0,06mm no trocantérico. A tensão máxima principal foi 10,4Pa, 7,52Pa e 26,4Pa nos grupos diafisário, subtrocantérico e trocantérico, respectivamente. Ou seja, a tensão máxima foi em torno de 40% maior no grupo trocantérico, em relação ao diafisário (controle). A face de tensão mínima do osso localizou-se na cortical interna do fêmur. A tensão equivalente de Von Mises nos implantes ocorreu na osteotomia, com valor máximo de 27,6Pa no grupo trocantérico.

**Conclusão** Tanto nas osteotomias no nível trocantérico, quanto subtrocantérico, a estabilidade da fixação foi muitas vezes menor que no modelo diafisário, sugerindo que as hastes intramedulares flexíveis não são implantes adequados para as fixações proximais do fêmur.

## Introdução

A fixação elástica intramedular é uma opção confiável e eficaz para o tratamento das fraturas situadas na diáfise do fêmur pediátrico. 1 2 No entanto, quando ocorre uma fratura nas regiões trocantérica ou subtrocantérica, o uso de hastes flexíveis é questionado devido a possível insuficiência da estabilidade mecânica para manter a redução e propiciar a consolidação. 3 4 5 6 Nestas condições é interessante simular o comportamento mecânico de um implante a fim de antecipar sua dependência ou não em condições clínicas.

Habitualmente, existem dois métodos de avaliar o comportamento mecânico do osso e implantes: técnicas experimentais diretas (ou métodos mecânicos) e modelos matemáticos. No entanto, as técnicas experimentais diretas apresentam desvantagens, sendo propensas a erros e imprecisões. 7

O método dos elementos finitos (MEF) é uma ferramenta poderosa desenvolvida inicialmente na década de 1950 e amplamente aceita após investimentos em tecnologia pela National Aeronautics and Space Administration (NASA). 8 No campo da engenharia, esse método é usado para resolver condições tão variadas quanto análise de tensões, fluxo de fluidos, eletromagnetismo e transferência de calor por modelos computacionais. 8

Na área médica, especialmente na ortopedia e biomecânica, os primeiros registros de aplicação do MEF remontam à década de 1970, quando foram realizadas estimativas da capacidade de diferentes tipos de ensaio na previsão do comportamento mecânico dos ossos. 7 9 Por meio do MEF, é possível obter uma representação precisa de geometrias complexas e incorporar as diferentes propriedades dos materiais, permitindo a aplicação de cargas em pontos específicos da estrutura. Dessa forma, é possível obter informações sobre as tensões máximas, mínimas e deformações. 7 Portanto, o MEF é utilizado para prever com precisão a resposta de um implante quando submetido a uma variedade de cargas, além de incorporar o efeito das interfaces entre o implante e o osso. 7 10 11

O objetivo do presente estudo foi avaliar a estabilidade fornecida por duas hastes intramedulares flexíveis em simulações de fraturas localizadas nas regiões subtrocantérica, trocantérica e diafisária criadas em um modelo de fêmur pediátrico, e utilizando o Método dos Elementos Finitos.

## Material e Métodos

Trata-se de um estudo realizado em laboratório utilizando modelos ósseos artificiais sendo, portanto, dispensada a aprovação do projeto pelo Comitê de Pesquisa Institucional.

Foi usado um modelo de fêmur infantil com dimensões correspondentes a uma criança de 9 anos de idade (Sawbone Inc., Pacific Research Laboratories Inc., WA, Estados Unidos). Esse osso sintético tem propriedades mecânicas semelhantes às do osso humano. 12 13

O preparo do espécime foi descrito anteriormente. 6 Em resumo, duas hastes flexíveis de titânio ( *Titanium Elastic Nail* - TEN®, TiGa 114v, DePuy Synthes®, Oberdorf, Suíça) com diâmetro de 3,5 mm foram inseridas retrogradamente no canal medular. Radiografias foram realizadas para confirmar o posicionamento adequado, seguidas por tomografia computadorizada de todo o modelo ósseo, que foram arquivadas no protocolo de comunicação DICOM ( *Digital Imaging and Communications in Medicine* ). A tomografia computadorizada foi realizada utilizando um tomógrafo Siemens® de 16 canais, modelo Emotion (Erlangen, Alemanha), com resolução de 512 × 512 e distância de corte de 1,0 mm. O DICOM foi importado para o programa InVesalius® (software livre do Centro de Tecnologia da Informação Renato Archer, Campinas, São Paulo, Brasil) no qual possibilitou a geração de modelos segmentados do sistema anatômico importado, para a construção tridimensional (3D) da estrutura anatômica. Uma vez obtido o objeto volumétrico reconstruído em três dimensões, o software permitiu a exportação do arquivo no formato Standard Triangle Language (STL).

A geração dos modelos virtuais 3D de cada conjunto osso-hastes foi realizada utilizando o programa Rhinoceros® 6 (Robert McNeel & Associates, Seattle, WA, Estados Unidos), versão 6. Para obter um contorno mais preciso e fiel, realizamos remodelações nas linhas de intersecção resultantes. Essas linhas foram traçadas com consideração à região em estudo, podendo conter variações na quantidade de pontos, conforme a necessidade de detalhes da área em questão. Em seguida, essas linhas foram interseccionadas e cortadas entre si, formando um conjunto de três ou quatro linhas. Esse conjunto permitiu a geração de uma superfície tridimensional.

A análise pelo Método dos Elementos Finitos (MEF) foi conduzida pelo programa SimLab® (HyperWorks, Troy, MI, USA), utilizando o solver Optistruct.

Para simular as fraturas, foram realizadas osteotomias nos modelos virtuais em três níveis: corte ao nível do trocanter menor (grupo trocantérico), corte localizado 3,5 cm distalmente ao trocanter menor (grupo subtrocantérico) e corte na região central da diáfise (grupo mediodiafisário, ou controle). Para a confecção das malhas, foram utilizados elementos tetraédricos, e definida a quantidade de nós. No ambiente virtual, um carregamento de 85,0 N foi aplicado no topo da cabeça do fêmur, na direção vertical, e obtidas as deformações e as tensões correspondentes.

Para as simulações foi necessário conhecer e definir as propriedades dos materiais de cada uma das partes dos modelos digitais, sendo eles: osso cortical, osso esponjoso e liga de titânio (TiGa114v). As propriedades dos materiais utilizados para as simulações estão apresentadas no Quadro 1 .

**Quadro 1:** Propriedades dos materiais nas simulações

Material	Propriedades	
	Módulo de elasticidade (MPa)	Coeficiente de Poisson (v)
Osso cortical	137	0,3
Osso esponjoso	13,7	0,3
Haste de titânio	114	0,33

## Resultados

Com um carregamento de 85,0 N foram obtidos os seguintes deslocamentos no local da simulação da osteotomia: 0,04 mm no grupo controle, 0,5 mm no grupo subtrocantérico e 0,06 mm no grupo trocantérico.

As maiores áreas de tensão foram identificadas na cortical lateral do fêmur e na região superior do colo. A tensão máxima principal atingiu valores de 10,4 Pa, 7,52 Pa e 26,4 Pa nos grupos controle, subtrocantérico e trocantérico, respectivamente ( Fig. 1 ).

**Fig. 1:** Distribuição das áreas de tensão máxima principal nas regiões proximais do fêmur nas simulações dos três tipos de osteotomias. A - Osteotomia diafisária, B - Osteotomia subtrocantérica, C - Osteotomia trocantérica. As cores vermelhas representam as áreas de maiores tensões.

A face de tensão mínima principal no osso foi identificada na cortical medial do fêmur, apresentando valores de -11,6 Pa no grupo controle, -9,95 Pa no grupo subtrocantérico e -25,9 Pa na osteotomia trocantérica. A tensão equivalente de Von Mises nos implantes foi observada na região da osteotomia, alcançando um valor máximo de 27,6 Pa no grupo trocantérico ( Fig. 2 ).

**Fig. 2:** A figura representa a reconstrução da região proximal do fêmur, o corte da osteotomia e as hastes flexíveis. No detalhe ao lado estão apresentadas as hastes sem o contorno ósseo, ilustrando a concentração das tensões equivalentes de Von Mises que são maiores na região da osteotomia (áreas em vermelho; região crítica). Se houver falência do implante ele vai ocorrer nesse nível, levando à perda da redução.

## Discussão

As fraturas que ocorrem na região subtrocantérica do fêmur apresentam forte tendência para o desvio do fragmento proximal em flexão, varo e rotação externa, associado ao encurtamento. 14 15 16 Isso resulta em aumento dos esforços entre os fragmentos, que se tornam mais dependentes do efeito estabilizador do implante.

Portanto, a fixação a ser utilizada deve ser capaz de neutralizar os momentos mecânicos gerados pelas forças locais, proporcionando estabilidade adequada para manter a redução e permitir a consolidação. Assim, as hastes elásticas podem não atender a esses critérios, como já mostram relatos clínicos 3 e ensaios mecânicos, 6 não sendo indicadas para fraturas nas regiões mais proximais.

Para estudar a estabilidade do conjunto modelo ósseo-implante, utilizamos o MEF, usado para simular e verificar a distribuição de tensão e deslocamentos a partir da solução de equações de equilíbrio sob cargas. 17 Para o uso da metodologia houve necessidade de usar um modelo de uma fratura, representado por uma osteotomia oblíqua. O MEF fornece os substratos teóricos e matemáticos, entretanto, em caso de fraturas, é aplicado a um modelo idealizado. Portanto, tem o inconveniente de não levar em consideração muitas características da fratura como irregularidades e diferentes inclinações do traço, além da possibilidade de apresentar mais de um fragmento. Além disso, não leva em consideração a ação das partes moles na estabilização/desestabilização da fratura. Essa limitação é inerente ao método, porém, mesmo com toda a simplificação, é muito útil nas avaliações pré-clínicas do desenvolvimento de um implante, por exemplo, o que é útil do ponto de vista de custo, tempo e de ética em pesquisa com seres humanos. Simplificações e restrições também ocorrem em estudos na área da engenharia e outras ciências exatas.

Há vários estudos envolvendo o MEF na ortopedia na literatura, sendo que os temas que envolvem fixação de fraturas e tratamento de lesões tumorais ósseas são os mais abordados. 11 18 19 20 21 22 O MEF, por não ser invasivo, fornece importantes informações biomecânicas, bem como auxilia no desenvolvimento de dispositivos ortopédicos e tem sido mais utilizado em modelos de estruturas anatômicas de adultos, inclusive para simulações de fixação de fraturas subtrocantéricas instáveis. 20 23 24 Wang et al. 20 avaliaram o desempenho biomecânico de três implantes para tratar fraturas subtrocantéricas instáveis em adultos usando o MEF, e observaram que a haste femoral proximal era mais estável que a haste bloqueada e o sistema LISS ( *Less Invasive Reverse System* ).

Nossos resultados mostraram que a osteotomia mais proximal (trocantérica) apresentou a maior tensão máxima e maior tensão equivalente de von Mises, o que indica que a demanda mecânica do implante é maior neste local do que nos outros dois grupos. Além disso, como a maior tensão equivalente a Von Mises ocorre nos locais das osteotomias, percebe-se que os implantes servem como “tutores” e protegem a fratura. Isso também foi observado no estudo de Soni et al. 25 que realizou uma simulação bidimensional em fraturas de fêmur em crianças com o MEF para avaliar a eficácia do uso de hastes flexíveis construídas em aço ou titânio.

Neste estudo, com a aplicação do carregamento, as regiões de maior tensão foram na cortical lateral do fêmur e na região superior do colo. Esses resultados mostram que, com a carga, o corte trocantérico apresentou solicitação de tensão 153% maior em comparação com o controle (corte mediodiafisário).

No entanto, o deslocamento dos fragmentos no local da osteotomia foi muito pequeno em todos os grupos, e isso pode ser atribuído ao baixo carregamento (85,0 N) aplicado aos sistemas. Este valor foi selecionado após levar em consideração a massa do membro inferior sem carga de uma criança de 10 anos (∼8,5 kg) 26 pois, clinicamente na fase pós-operatória precoce não se recomenda a aplicação de carga intencional. Adicionalmente, este carregamento permitiu que a deformação ficasse restrita à fase elástica dos implantes, ou seja, que não houvesse deformação irreversível, o que ocorre na clínica. Se esse limite for ultrapassado haverá deformação permanente do implante e perda da redução da fratura.

## Conclusão

Para as osteotomias nas regiões trocantérica e subtrocantérica, há solicitação mecânica maior do implante, que pode ultrapassar os limites de estabilização das hastes intramedulares flexíveis. Assim, clinicamente, esse tipo de implante deve ser indicado nas situações clássicas para que foi concebido, isto é, nas fraturas da região diafisária do fêmur.
